# InCHlib – interactive cluster heatmap for web applications

**DOI:** 10.1186/s13321-014-0044-4

**Published:** 2014-09-17

**Authors:** Ctibor Škuta, Petr Bartůněk, Daniel Svozil

**Affiliations:** 1 Laboratory of Informatics and Chemistry, Faculty of Chemical Technology, Institute of Chemical Technology Prague, Technická 5, Prague, CZ-166 28 Czech Republic; 2 CZ-OPENSCREEN, Institute of Molecular Genetics of the ASCR, v. v. i, Vídeňská 1083, Prague, CZ-142 20 Czech Republic

**Keywords:** Data clustering, Cluster heatmap, Scientific visualization, Web integration, Client-side scripting, JavaScript library, Big data, Exploration

## Abstract

**Background:**

Hierarchical clustering is an exploratory data analysis method that reveals the groups (clusters) of similar objects. The result of the hierarchical clustering is a tree structure called dendrogram that shows the arrangement of individual clusters. To investigate the row/column hierarchical cluster structure of a data matrix, a visualization tool called ‘cluster heatmap’ is commonly employed. In the cluster heatmap, the data matrix is displayed as a heatmap, a 2-dimensional array in which the colour of each element corresponds to its value. The rows/columns of the matrix are ordered such that similar rows/columns are near each other. The ordering is given by the dendrogram which is displayed on the side of the heatmap.

**Results:**

We developed *InCHlib* (Interactive Cluster Heatmap Library), a highly interactive and lightweight *JavaScript* library for cluster heatmap visualization and exploration. *InCHlib* enables the user to select individual or clustered heatmap rows, to zoom in and out of clusters or to flexibly modify heatmap appearance. The cluster heatmap can be augmented with additional metadata displayed in a different colour scale. In addition, to further enhance the visualization, the cluster heatmap can be interconnected with external data sources or analysis tools. Data clustering and the preparation of the input file for *InCHlib* is facilitated by the Python utility script *inchlib_clust*.

**Conclusions:**

The cluster heatmap is one of the most popular visualizations of large chemical and biomedical data sets originating, e.g., in high-throughput screening, genomics or transcriptomics experiments. The presented *JavaScript* library *InCHlib* is a client-side solution for cluster heatmap exploration. *InCHlib* can be easily deployed into any modern web application and configured to cooperate with external tools and data sources. Though *InCHlib* is primarily intended for the analysis of chemical or biological data, it is a versatile tool which application domain is not limited to the life sciences only.

**Electronic supplementary material:**

The online version of this article (doi:10.1186/s13321-014-0044-4) contains supplementary material, which is available to authorized users.

## Background

Clustering is a data exploration technique that identifies groups of objects that are similar to each other but different from objects in other groups [1]. Cluster analysis is widely applied in cheminformatics for the analysis of databases of chemical structures [2],[3]. Its main use is to find representative subsets from high throughput screening (HTS) [4]-[6], to design chemical libraries of diverse structures pertinent to pharmaceutical discovery [7]-[9] and to increase the diversity of these libraries through the selection of additional compounds from other data sets [10],[11]. The most popular approach of cluster analysis is hierarchical clustering [12] in which data are merged together based on a tree structure called dendrogram. The input to a clustering algorithm is a data matrix that contains individual data points in rows and data features in columns. Data can be clustered either by rows or by columns. The data matrix can be visualized as a ‘data heatmap’, a rectangular array that uses colour to represent numerical values of individual matrix cells. The data heatmap augmented with row and/or column dendrograms is known as a ‘cluster heatmap’ [13],[14].

Owing to the wide application of the cluster heatmap in biomedical sciences [15], many software tools for its visualization and exploration are available. Several of them, such as the *R* programming environment [16] with *Bioconductor* package [17], *CIMminer*[18] or *Cluster/TreeView*[19],[20], generate only static images with fixed appearance and no interactivity. Higher level of interactivity offer standalone programs typically implemented in *Java* programming language that are, however, usually tailored towards the analysis of specific data [21],[22]. For example, the following packages enable the analysis of gene expression experiments: *Java Treeview*[23], *High-Throughput GoMiner*[24], *TM4*[25], *Genesis*[26] or *PageMan*[27]. Similarly, genomics data can be explored by *geWorkbench*[28], *StratomeX*[29], *GENE-E*[30], *Qcanvas*[31] or *Gitools*[32]. The main disadvantage of desktop solutions is their limited set of features that cannot be easily enhanced by the user. In addition, desktop applications cannot be readily deployed in modern web-based systems.

In recent years, client-side scripting became very popular for the development of interactive web solutions. The client is the system on which the web browser runs. Client-side scripts are interpreted by the browser and they work in the following steps: (1) the user requests the web page from the server, (2) the server finds the page and sends it to the user, (3) the page is displayed in the browser with any scripts run during or after display. Because all data processing is performed by the client, the speed of the script execution depends on the user’s hardware. Two types of clients exist: thick (fat) and thin clients. The thick clients are written in full-blown programming languages, such as *Java* or *C#*. To be executed, thick clients require additional software (e.g., *Java Virtual Machine* or *.NET framework*) to be installed on the user machine. On the other hand, the thin client is executed by an engine embedded directly in the web browser. The main scripting language for the thin client programming is *JavaScript. JavaScript* is a powerful, easy to learn and use language which became an integral part of many existing web technologies. Compared to the thick client, the thin client typically requires less performing user devices equipped with lower amounts of memory.

If the deployment of the cluster heatmap into a web application is required, possibilities are rather limited. Though several web solutions for the analysis of genomics data exist, such as The *UCSC Cancer Genomics Browser*[33],[34], *Expression Profiler*[35], *Babelomics*[36], *Next-Generation Clustered Heatmaps*[37] or INVEX [38], they work as standalone web applications. It means that they can be used only from their hosting websites and their interface reflects the nature of the data they are designed to work with. The use of such applications for the analysis of, often sensitive, user’s data requires the data to be uploaded to the web server of the application provider. Though a few thick clients exist (e.g., *Gitools*[32]), the availability of *JavaScript* solutions for cluster heatmap exploration is rather limited. While *jHeatmap*[39] and the *BioJS HeatmapViewer* component [40] can display only the data heatmap without its underlying cluster structure, the Heatmap viewer from the *JavaScript* library *canvasXpress*[41] offers only limited functionality. Thus, we developed *InCHlib*, a free browser independent *JavaScript* library that facilitates the visualization, exploration and web integration of the cluster heatmap. Though *InCHlib* is primarily intended for the analysis of chemical or biological data, its application domain is not limited to the life sciences only.

## Implementation

*InCHlib* is a free browser independent *JavaScript* library which *HTML5* canvas-based rendering is handled by the *KineticJS*[42] framework (version 5.0.0) and *HTML* elements are processed using the *jQuery* framework [43] (version 2.0.3). *InCHlib* enables to interact in real time with other elements on the page or with external data sources. This is achieved by handling the events that occur during the user interaction with the cluster heatmap, such as clicking on a heatmap row or dendrogram node. For each event, a callback function can be defined and invoked if the event is triggered. The interconnection between the visualization and external data sources is realized by the exchange of the IDs of passed objects. The tutorial with commented examples demonstrating all steps of *InCHlib* deployment is available at http://openscreen.cz/software/inchlib/examples/18.

### Input format

*InCHlib* is a visualization library and is, thus, not responsible for data clustering. Instead, data must be clustered by an external program, such as *inchlib_clust* (see the ‘inchlib_clust’ paragraph) and then passed into *InCHlib* either as a *JavaScript* variable or as a file stored in the *InCHlib* input format. The *InCHlib* input conforms the *JSON* (*JavaScript Object Notation*) standard [44]. Key elements of the *InCHlib* input format are demonstrated by the code snippets in this section and the complete example of the input file is given in Additional file 1.

**Figure 1:**
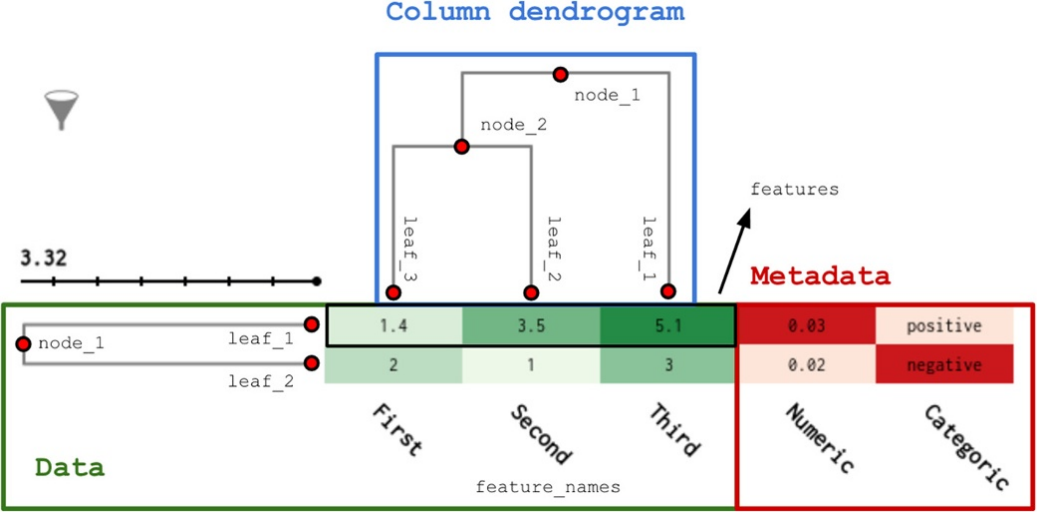
**An example cluster heatmap.** The visualization consists of three blocks described in the *InCHlib* input format. The data block (green) contains feature names and the data for the rendering of the row dendrogram and heatmap. The metadata block (red) contains the additional data that are appended to the original data after clustering. The column dendrogram block (blue) contains the data for the rendering of column dendrogram.

The input format describes three parts cluster heatmap visualization consists of: *data, metadata* and *column dendrogram* (Figure 1). The *data block* contains the data matrix and describes the structure of the row dendrogram. The row dendrogram consists of inner and terminal (usually referred to as leaves) nodes connected by branches. Each leaf is associated with one ‘data item’, i.e., with one heatmap row. Each data item corresponds either to one data point or, if the row reduction is used (see further), to several data points merged into one. Each data item is annotated with the IDs of data points it comprises of. The following code snippet demonstrates how the leaf is described in the *InCHlib* format.

"leaf_1": { //ID of the node (leaf)

"count": 1, *//number of objects (heatmap rows) which lie in the dendrogram hierarchy below the given node*

"distance": 0, *//distance in dendrogram measured from leaves to the root node given by the distance measure used for the clustering*

"features": [1.4, 3.5, 5.1], *//values of individual features forming a data item*

"parent": "node_1", *//the ID of a parent node*

"objects": ["object_1", “object_2”] *// IDs of data points represented by the given row*

},

Each node is identified by a unique ID string. While each inner node has two children, no child exists for the leaf. Children of a node are given as the *left_child* and *right_child* parameters. ID of the parent’s node is given as the *parent* parameter. The only node without the *parent* is the root node of the dendrogram. The following code snippet demonstrates how the node is described in the *InCHlib* format.

"node_1": { //ID of the node

"count": 3, *//number of objects (heatmap rows) which lie in the dendrogram* hierarchy below the given node

"distance": 3.32, *//distance from the zero base of the dendrogram,* given by the distance measure used for clustering

"parent": "node_1", *//the ID of a parent node*

"left_child": "leaf_1", *//ID of a left child*

"right_child": "leaf_2" *//ID of a right child*

},

The *metadata block* (Figure 1) describes additional information associated with individual data items, such as class membership. The metadata, displayed as additional column(s) in the heatmap, have no influence on the order of data items because they are not subjected to the clustering. The following code snippet shows how the metadata are described in the *InCHlib* format.

"metadata": { *//contains nodes and feature_names section of metadata*

"feature_names": ["Numeric", "Categoric"], *//names of metadata features*

"nodes": { *//contains object IDs with metadata features*

"leaf_1": [0.03, "positive"], *// metadata features*

"leaf_2": [0.02, "negative"]

}

},

The *column dendrogram block* (Figure 1) of the *InCHlib* input format describes the vertical dendrogram and has the same structure as the row dendrogram. The only difference is that leaves don't have the *features* and *objects* parameters *inchlib_clust*.

To facilitate the preparation of data in the *InCHlib* format, we developed a utility script *inchlib_clust. inchlib_clust* is written in *Python 2.7* programming language. It performs both data preprocessing, such as data normalization or compression, and hierarchical clustering. Hierarchical clustering in *inchlib_clust* is accomplished by the *fastcluster*[45] library that implements several common hierarchical clustering schemes. List of available *fastcluster* linkages and distances is given in Additional file 2. Clustering results are saved in the *InCHlib* input file that can be readily passed into *InCHlib. inchlib_clust* can be easily extended by other hierarchical clustering approaches, such as by the popular Super Paramagnetic Clustering (SPC) [46],[47] which scales more favourably (as *O(N)*) than the *O(N*^*2*^*)* implementation of hierarchical clustering in *fastcluster*.

Data normalization is a preprocessing step used to balance the influence of features measured at different scales. *inchlib_clust* enables features to be scaled to the range between 0 and 1 using the *MinMax* scaler. *MinMax* scaler transforms the original feature *x* into its normalized version *x* ' according to the formula

$$x^{'}=\frac{x-\min\left(x\right)}{\max\left(x\right)-\min\left(x\right)}$$

where min(*x*) and max(*x*) are minimum and maximum values of the feature *x*. If the data normalization is used, the order of the heatmap rows (i.e., the row dendrogram) is always given by the clustering of the normalized data. However, the user can choose whether the normalized or original data will be displayed in the heatmap.

Because the speed of rendering decreases as the number of rows increases (see the ‘Performance assessment’ paragraph), *inchlib_clust* also enables to reduce the size of the data matrix. To increase the speed of visualization, as well as to reveal new data motifs by noise suppression, the number of the data matrix rows can be reduced. In row reduction, similar rows are aggregated into a single vector. Elements of this vector are calculated as the mean or median values of the elements of original rows. The extent of the compression is given as the number of reduced data matrix rows.

Another possibility how to speed up the visualization is to completely hide the data heatmap. In such case, only the dendrogram and metadata are displayed. This option comes in handy when the number of dimensions (columns) is too high, such as in the case of hashed chemical fingerprints.

## Results and discussion

In this section, a typical *InCHlib* use consisting of data preparation and web page deployment is described. In addition, advanced *InCHlib* capabilities are demonstrated on the clustering of the ligands of *estrogen receptor* α (*ER*α). Finally, the speed of both data clustering by *inchlib_clust* and data visualization by *InCHlib* is evaluated.

**Figure 2:**
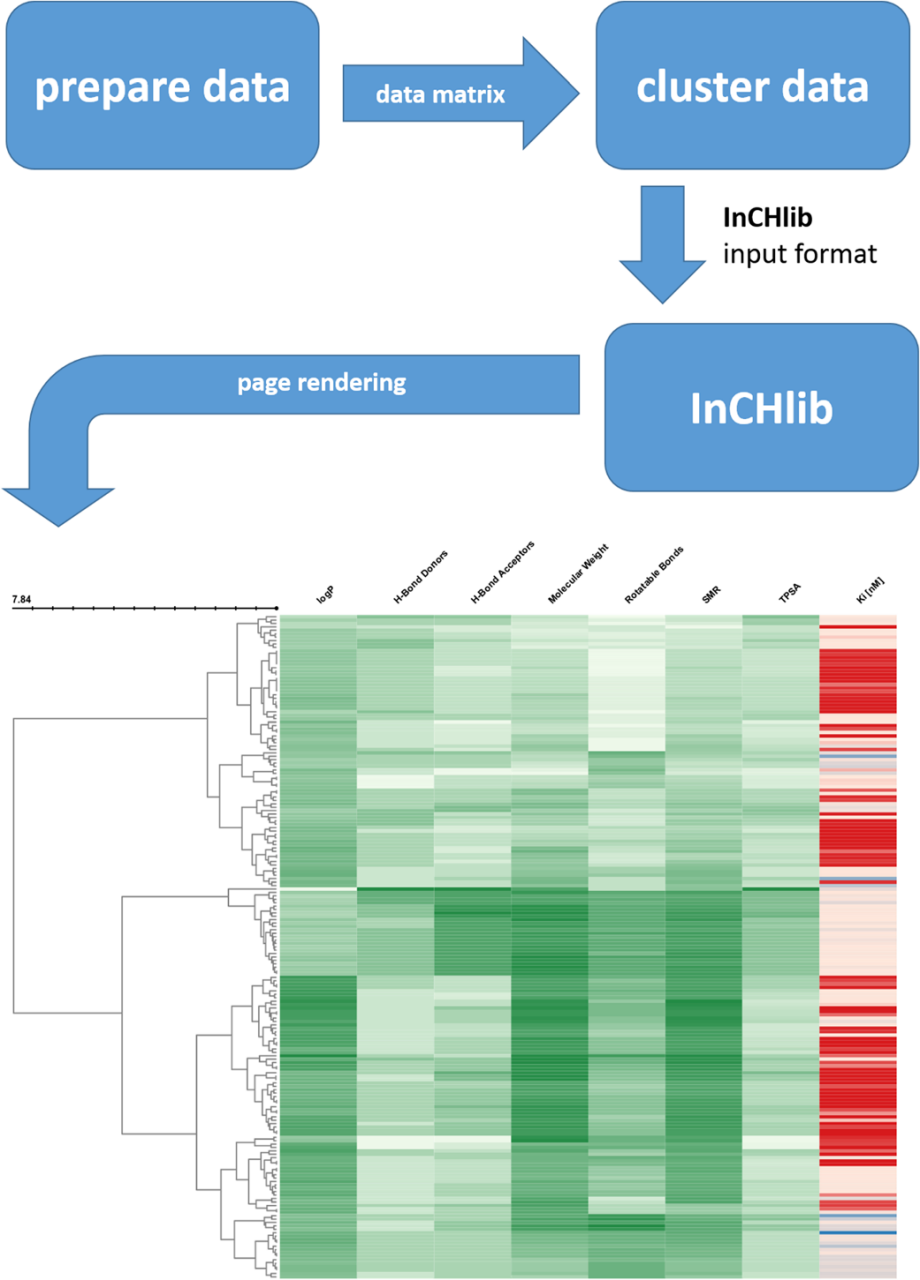
**The InCHlib deployment pipeline.** It consists of three steps: data preparation, clustering and rendering. In the data preparation step, the data matrix consisting of data points and their IDs in rows and their features in columns is stored in the text file. Similarly, metadata are saved in a separate text file. In the clustering step, these files are supplied to the software that performs hierarchical clustering and stores the results in the *InCHlib* input file. Though any clustering software can be used, a utility script *inchlib_clust* that uses *fastcluster* library for clustering and outputs data directly in the *InCHlib* format was developed. In the third step, the *InCHlib* input file is read in by *InCHlib* which renders the cluster heatmap visualization.

The deployment of *InCHlib* consists of several steps (Figure 2): data preparation, data clustering, web page integration and cluster heatmap visualization.

Though data can be clustered by *inchlib_clust*, any clustering software can be used provided that the valid *InCHlib* input file is generated. Typically, the data matrix is supplied to *inchlib_clust* in a comma-separated values (*csv*) file, though other delimiters, such as tab or semicolon, are also possible. The data matrix consists of data points in rows and their features in columns. The first column always contains the IDs of individual rows. Optionally, feature names are given in the first row. The example of the data file is given in Additional file 3. Similarly, metadata are supplemented as a separate file using the same format. More metadata columns can be specified, and the metadata can be both numerical (e.g., *EC50*) or categorical (e.g., class membership). The metadata are associated with the corresponding data through their respective IDs. The example of the metadata file is given in Additional file 4.

### Clustering

The only mandatory input to *inchlib_clust* is the data matrix stored in the *csv* file. If default parameters are used, no data scaling or row compression is applied and the data are clustered by rows using Ward’s clustering with the Euclidean distance. For example, to cluster the data stored in the *example_data.csv* file using the Ward’s clustering with the Euclidean distance, the following command line is used:

python inchlib_clust.py example_data.csv –m example_metadata.csv -dh –mh –a both -o example.json

In this case, the metadata are supplied (option *-m*) as the *example_metadata.csv* file, and both data and metadata contain column headers (−*dh* and *-mh* options). The data are clustered both by rows and columns (−*a both* option). The output file *example.json* (Additional file 1) contains the cluster heatmap in the InCHlib input format. Besides the command line interface, *inchlib_clust* also offers the application programming interface (*API*) and can, thus, be invoked from the user code. The use of *inchlib_clust API* from the *Python* script is demonstrated in Additional file 5.

Once the *InCHlib* input file is created, it is read by *InCHlib* and the cluster heatmap is visualized. Prior calling *InCHlib* functions, *KineticJS* and *jQuery* libraries must be imported. Then, the *InCHlib* object is instantiated with the *settings* parameter (given as the JavaScript object), the *JSON* file is read using the *read_data_from_file()* method and the cluster heatmap is rendered by calling the *draw()* method. The only obligatory attribute of the *settings* parameter is the *target* attribute that defines the *id* of the *HTML* element the cluster heatmap is inserted in. Other optional attributes of the *settings* parameter influence the appearance of the visualization (e.g., *colors* or *size* attributes) or of its individual parts (e.g., *row dendrogram, column dendrogram, heatmap* or *metadata* attributes). The example of the *HTML/JavaScript* code demonstrating InCHlib web page integration is given in Additional file 6. The resulting web page with commented *HTML/JavaScript* code is shown in Additional file 2.

### Use case

In this section, the use of *InCHlib* for the exploration of the *estrogen receptor* α (*ER*α) ligand binding will be demonstrated. *ER*α belongs to the family of steroid hormone receptors [48], ligand-inducible transcription factors that control essential physiological, developmental, reproductive and metabolic processes [49],[50]. *ERs* are overexpressed in around 70% of breast cancer cases [51] and have also been implicated in ovarian, colon and prostate cancers. Thus, *ERs* represent an important target for therapeutic intervention [52].

The analysed data consist of 8 physico-chemical and structural properties of 195 *ER*α ligands obtained from the *ChEMBL* database [53]. The ligand properties were calculated by the *RDKit* cheminformatics toolkit [54] and they include the logarithm of the octanol-water partition coefficient (*logP*), molar refractivity (*SMR*), topological polar surface area (*TPSA*), molecular weight, and number of rotatable bonds, hydrogen-bond donors, hydrogen-bond acceptors and aromatic rings. To each ligand, its metadata represented by the *K*_*i*_ value (equilibrium dissociation constant determined in inhibition studies) is also assigned.

**Figure 3:**
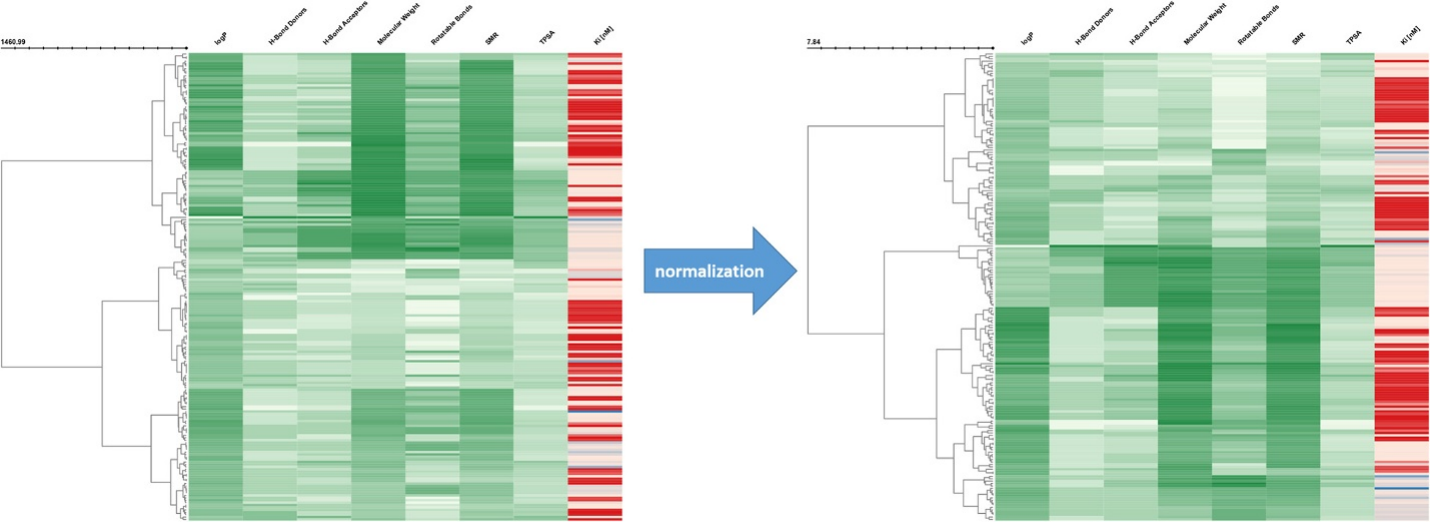
**The comparison of ERα** **ligand clusterings performed with original (left panel) and normalized (right panel) values.** The data were clustered by Ward’s clustering with the Euclidean distance. In the case of the clustering of normalized data (right panel), original data values are depicted in the heatmap (parameter *--write_original* of *inchlib_clust*).

The results of the hierarchical Ward’s clustering with the Euclidean distance performed by *inchlib_clust* are shown in the left panel of Figure 3. In this heatmap, physico-chemical properties and *K*_*i*_ show no clear relationship. However, the clustering is biased by the wide range of molecular weight (250 – 600 Da). Because the values of other features are from narrower intervals (e.g., *logP* has values between 3 and 6), molecular weight prevails and the data are clustered mainly by this descriptor. To remove this artefact, data were normalized to the scale between 0 and 1. After the normalization, the data became more ordered (Figure 3, right panel) and correlated with *K*_*i*_ values. Such patterns indicate potential relationships between physico-chemical descriptors and biological activity.

**Figure 4:**
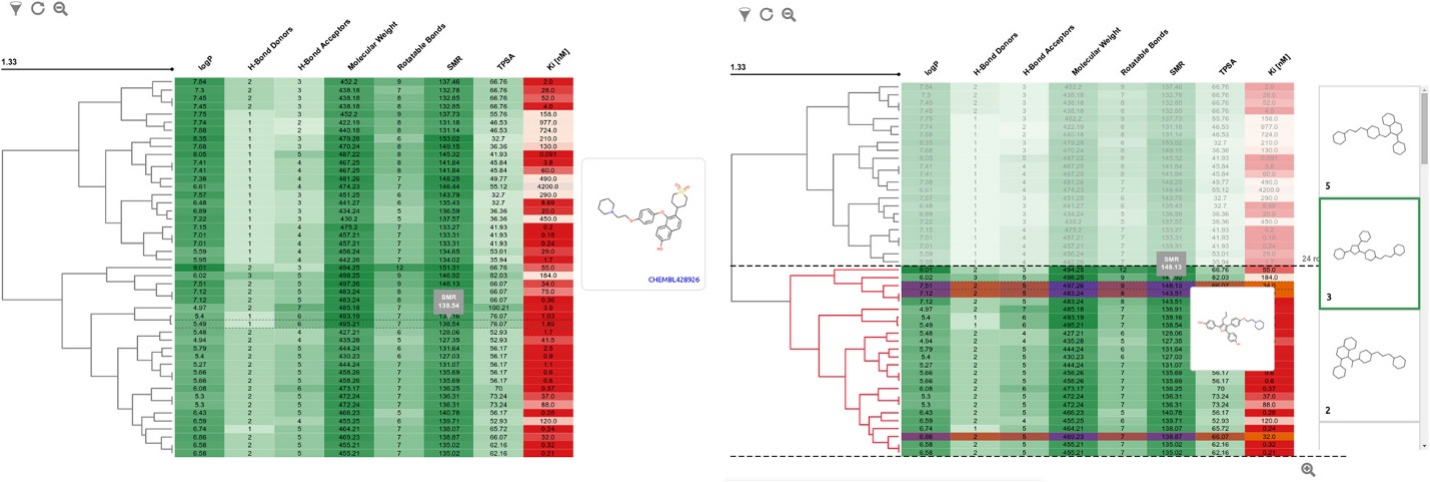
**The cluster heatmap of ERα** **ligands enhanced by the structure visualization.** On the left panel, the *row_onmousover* event is used for the visualization of molecular structure. On the right panel, the scaffold composition of the selected cluster is shown by handling the *dendrogram_node_onclick event*. If the scaffold image is clicked, the heatmap rows representing compounds with the selected scaffold are highlighted in red.

To facilitate the discovery of the structure-activity relationships, depictions of ligand structures are shown right of the cluster heatmap (Figure 4, left panel). This is achieved by handling the *row_onmouseover* event. This event is triggered upon hovering the mouse over the row and displays the ligand image. The ligand image, which is pre-generated by the chemoinformatics toolkit *RDKit*[54], is stored in the file *CHEMBLID*.png. For example, *CHEMBL1276308.png* contains the structure of mifepristone, the compound with the *CHEMBL1276308* ID. The structure depiction is hyperlinked with the *CHEMBL* database and, upon clicking the structure image, the corresponding *CHEMBL* record opens in a new tab.

Though the depiction of molecular structures is useful, the next step in the discovery of structure-activity relationships is the so-called scaffold analysis. Molecular scaffold is the graph representation of a molecular core structure [55]. Molecular scaffolds were successfully applied, among other, to the diversity analysis [56],[57] of bioactive compounds [58]-[64]. In the *ER*α use case, molecular scaffolds are revealed when the cluster is selected (Figure 4, right panel). This is achieved by handling the *dendrogram_node_onclick* event. When the scaffold image is clicked, compounds with the given scaffold are highlighted (Figure 4, right panel)*.* The colour of highlighted rows is set as a *highlight_colors settings* attribute on *InCHlib* instantiation; the default colour scheme is *Reds*. In the presented use case, scaffolds of all 195 ligands are extracted and their images are generated by the *RDKit*[54] toolkit. A unique ID is assigned to each scaffold and scaffold image is stored in the *ID.png* file. To display the scaffold images upon node clicking, we implemented the server-side Python function that accepts the list of compound IDs (*CHEMBL IDs*), extracts the molecular scaffold of each compound and groups the compounds with the identical scaffolds. The function returns an array of scaffold IDs with attached compound IDs. For example, the array *[1, ["CHEMBL234638", "CHEMBL278703", "CHEMBL234633"]]* contains 3 compounds that share a common scaffold with ID *1*.

The *ER*α use case, as well other examples demonstrating the use of *InCHlib* for the exploration of protein structures, identification of gene expression patterns or classification of whiskies based on their taste characteristics, are available from http://openscreen.cz/software/inchlib/use_cases/13. In addition, their short description is given in Additional file 2.

### Performance assessment

To assess the performance of *inchlib_clust* and *InCHlib*, the dependence of the speed of clustering (*inchlib_clust*) and rendering (*InCHlib*) on the data size was investigated. The data, consisting of randomly generated integers between 0 and 1 000, were clustered using the Euclidean distance and Ward’s linkage. Experiments were performed using the following computer configuration: *Kubuntu 13.10*, *Chrome 33.0.1750.146*, Intel Core i5-2400 CPU 3.10 GHz, 8 GB RAM, 120 GB solid-state drive (SSD).

**Figure 5:**
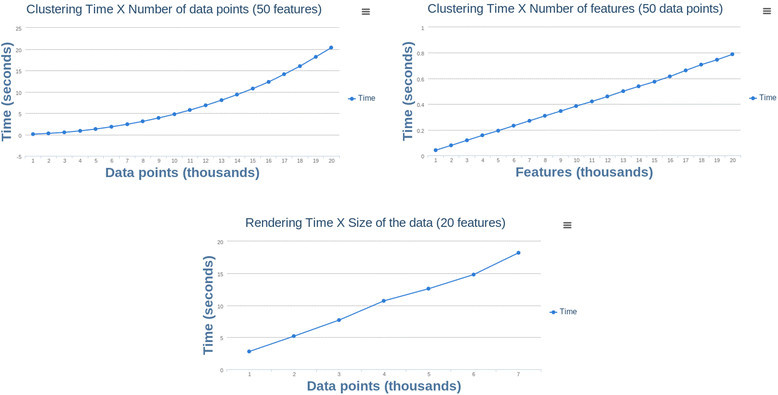
**The speed of clustering and heatmap rendering.** Top panel: the dependence of the speed of clustering by *inchlib_clust* on the number of data points (i.e., the number of data matrix rows) and on the number of features (i.e., the number of data matrix columns). Bottom panel: the dependence of the speed of rendering by *InCHlib* on the data size.

Clustering time increases quadratically with the number of data points (Figure 5, top left panel) which corresponds to the *O(N*^*2*^*)* complexity of the implementation of the Ward linkage hierarchical clustering in the *fastcluster* library [45]. Similarly, memory requirements increase with the number of data points; while clustering of 10,000 data points required 0.5 GB of RAM, memory consumption grew up to 2 GB for clustering of 20,000 data points. Contrary to the quadratic increase in clustering time with the increase of the number of data points (i.e., rows of the data matrix), the dependence of the clustering speed on the number of features (i.e., columns of the data matrix) is linear (Figure 5, top right panel).

In addition to the performance of *inchlib_clust*, speed of *InCHlib* rendering was also investigated. *InCHlib* rendering time depends linearly on the number of data points (Figure 5, bottom panel). While the linear dependence is the feature of the *InCHlib* implementation, absolute rendering times are greatly influenced by the PC hardware and web browser in which the primary limiting factor is the speed of the *JavaScript* engine.

## Conclusions

*InCHlib* is a browser independent *JavaScript* library that facilitates the uncluttered visualization, powerful exploration and easy web integration of the cluster heatmap. *InCHlib* is an interactive tool that enables the user to select individual or clustered heatmap rows, to zoom in and out of clusters or to flexibly modify heatmap appearance. The *InCHlib* application programming interface defines a rich set of events through which the visualization can be interconnected with external data sources and analysis tools. The cluster heatmap can be augmented with additional metadata displayed in a different colour scale. To reduce the size of the heatmap and to reveal unique motifs in the data, number of rows can be limited by using several averaging methods. The clustered data are passed into *InCHlib* in a *JSON* compliant input data format. To facilitate data clustering and *InCHlib* input preparation, the Python utility script *inchlib_clust* can be employed. Though *InCHlib* is primarily intended for the analysis of chemical or biological data, its application domain is not limited to the life sciences only. *InCHlib* has already been successfully deployed at the *Institute of Molecular Genetics AS CR* as the part of an high-throughput screening information management system used at *CZ-OPENSCREEN: National Infrastructure for Chemical Biology. InCHlib* and *inchlib_clust* are provided free for download, and *InCHlib* is also available as the *BioJS*[65] component.

## Availability and requirements

**Project name:** InCHlib

**Project home page:**http://openscreen.cz/software/inchlib/home/, https://www.ebi.ac.uk/Tools/biojs/registry/Biojs.InCHlib.html

**Operating system(s):** platform independent

**Programming language:** JavaScript

**Other requirements:** Python 2.7 to run *inchlib_clust*

**License:** MIT

**Any restrictions to use by non-academics:** None

## Authors’ contributions

DS and PB instigated the project, participated in the development of the software, collected data sets, proposed the use cases and drafted the manuscript. CŠ is the lead developer of *InCHlib*. He designed and implemented the application and all use cases, prepared the web pages, performed all tests and calculations and helped to draft the manuscript. All authors read and approved the final manuscript.

## Additional files

## Electronic supplementary material

Additional file 1: **Commented JSON** ***InCHlib*** **input file.**(ZIP 829 bytes)

Additional file 2: **Supplementary information with** ***inchlib_clust*** **clustering options and use cases.**(PDF 456 KB)

Additional file 3: Example data file.(CSV 65 bytes)

Additional file 4: Example metadata file.(CSV 74 bytes)

Additional file 5: **Commented example of the use of the** ***inchlib_clust*** **application programming interface.**(ZIP 765 bytes)

Additional file 6: **Example of the integration of** ***InCHlib*** **into a web page.**(ZIP 424 bytes)

Below are the links to the authors’ original submitted files for images.

Authors’ original file for figure 1

Authors’ original file for figure 2

Authors’ original file for figure 3

Authors’ original file for figure 4

Authors’ original file for figure 5
