# Supplementary material for: InCHlib – interactive cluster heatmap for web applications
Source: J Cheminform. 2014 Sep 17;6:44. doi: 10.1186/s13321-014-0044-4 (PMC4173117; doi:10.1186/s13321-014-0044-4)
Supplement: Supplementary file 2 — Additional file 2: Supplementary information with inchlib_clust clustering options and use cases.(PDF 456 KB) [file 13321_2014_44_MOESM2_ESM.pdf]

Supplementary information

InCHlib – Interactive Cluster Heatmap for web applications

Škuta C.[1,2], Bartůněk, P.[2] and Svozil, D.[1,2*]

[1] Laboratory of Informatics and Chemistry, Faculty of Chemical Technology, Institute of Chemical Technology Prague, Technická 5, CZ-166 28 Prague, Czech Republic

[2] CZ-OPENSCREEN, Institute of Molecular Genetics of the ASCR, v. v. i., Vídeňská 1083, CZ-142 20 Prague, Czech Republic

Contact: svozild@vscht.cz

1. *inchlib_clust* clustering options

inchlib_clust is a Python script that performs data clustering and prepares input data for *InCHlib*. *inchlib_clust* can be used both from the command line or *Python* code. Its documentation and use examples are available from the *InCHlib* website [1]. *inchlib_clust* performs a hierarchical clustering using *fastcluster* [2] library. The following linkages and distance measures are available.

Linkages:

1. single - the single/min/nearest algorithm
2. complete - the complete/max/farthest algorithm
3. average - the average/UPGMA algorithm
4. weighted - the weighted/WPGMA algorithm
5. centroid - the centroid/UPGMC algorithm
6. median - the median/WPGMC algorithm
7. ward - the Ward/incremental algorithm

Distance measures:

1. braycurtis - the Bray-Curtis distance
2. canberra - the Canberra distance
3. chebyshev - the Chebyshev distance
4. cityblock - the Manhattan distance
5. correlation - the Correlation distance
6. cosine - the Cosine distance
7. dice - the Dice dissimilarity (boolean)
8. euclidean - the Euclidean distance
9. hamming - the Hamming distance (boolean)
10. jaccard - the Jaccard distance (boolean)
11. kulsinski - the Kulsinski distance (boolean)
12. mahalanobis - the Mahalanobis distance
13. matching - the matching dissimilarity (boolean)
14. minkowski - the Minkowski distance
15. rogerstanimoto - the Rogers-Tanimoto dissimilarity (boolean)
16. russellrao - the Russell-Rao dissimilarity (boolean)
17. seuclidean - the normalized Euclidean distance
18. sokalmichener - the Sokal-Michener dissimilarity (boolean)
19. sokalsneath - the Sokal-Sneath dissimilarity (boolean)
20. sqeuclidean - the squared Euclidean distance
21. yule - the Yule dissimilarity (boolean)

2. *InCHlib* deployment

In this section, a short example demonstrates how to integrate *InCHlib* into a web page. The commented *HTML/JavaScript* code is followed by the rendered cluster heatmap. The input data file example.json is given in Additional file 1.

```
<html>
    <head>
        <script src="/path/to/jquery-2.0.3.min.js"></script> //import jQuery
        <script src="/path/to/kinetic-v5.0.0.min.js"></script> //import KineticJS
        <script src="/path/to/inchlib-1.0.0.js"></script> //import InCHlib
        <script>
        $(document).ready(function() { //execute when the web page is ready
            var inchlib = new InCHlib({ //InCHlib instantiation
                target: "inchlib", //define target element
                metadata: true, //input file contains metadata
                column_dendrogram: true, //input file contains column dendrogram
            });

            inchlib.read_data_from_file("/path/to/example.json"); //read data from file
            inchlib.draw(); //draw cluster heatmap
        });
        </script>
    </head>

    <body>
        <div id="inchlib"></div> //target element
    </body>
 </html>
```

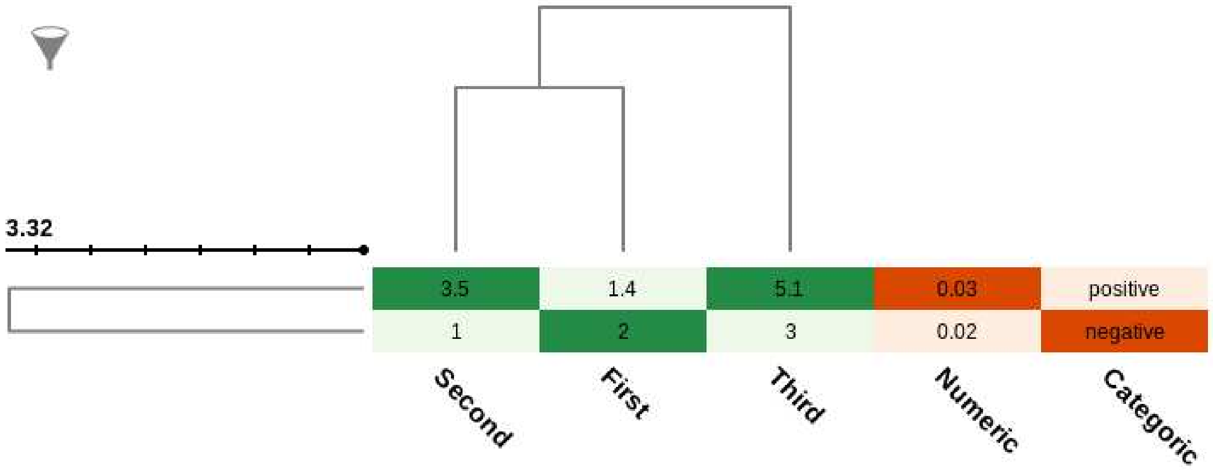

3. Use examples

In this section, various use cases demonstrating *InCHlib's* versatility are presented.

3.1. Chemical biology data

InChlib use in chemical biology is demonstrated by this example. The data set consists of 8 physico-chemical and structural properties of 195 *estrogen receptor alpha (ERα)* ligands obtained from the *ChEMBL* database [3]. The ligand properties are: the logarithm of the octanol-water partition coefficient (*logP*), molar refractivity (*SMR*), topological polar surface area (*TPSA*), molecular weight, and number of rotatable bonds, hydrogen-bond donors, hydrogen-bond acceptors and aromatic rings. Each ligand is also characterized by its K_i value (equilibrium dissociation constant determined in inhibition studies) that is considered as the medata.

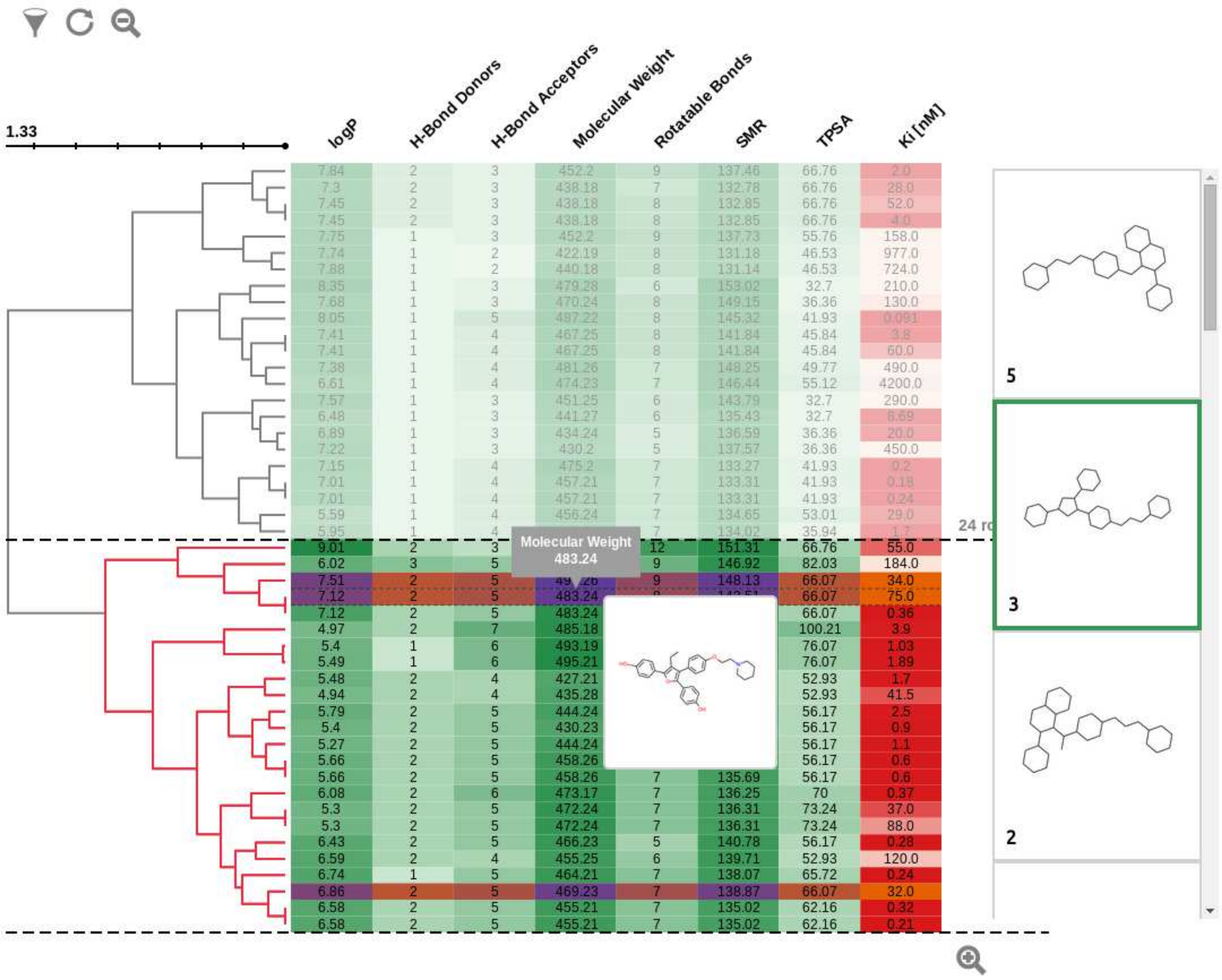

3.2. 3D protein structure

This example demonstrates how to combine *InCHlib* with 3D protein structure visualization. Each protein is characterized by the percentage of its 20 amino acid (green data matrix). There are no metadata in this example. 3D structure visualization is delivered by the *GLmol 3D viewer* [4]. Various information about the structure with links to external databases are summarized in the table below the 3D visualization.

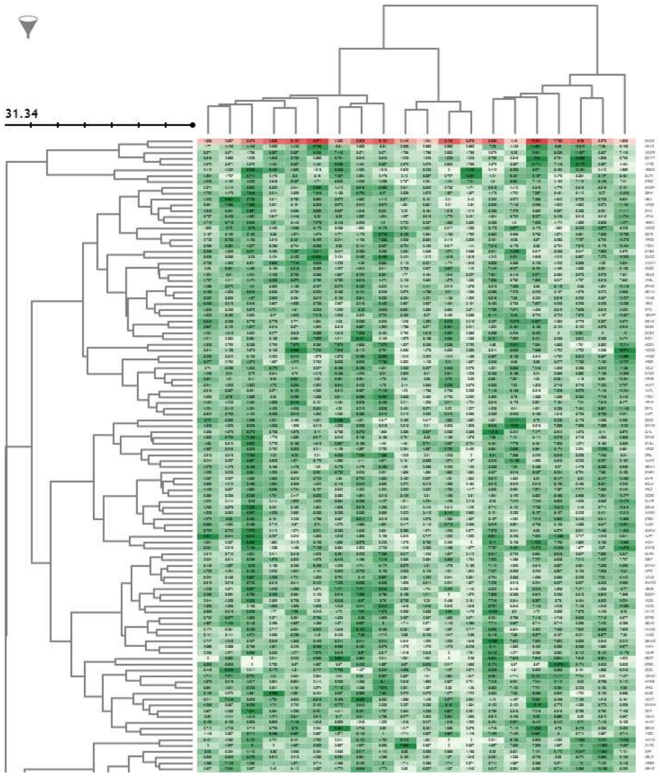

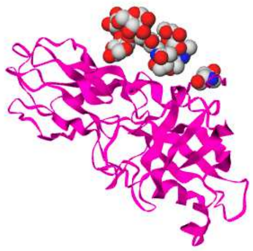

**PDB ID**	2HCZ
**Structure Title**	Crystal structure of EXPB1 (Zea m 1), a beta-expansin and group-1 pollen allergen from maize
**Chain Length**	245
**Resolution**	2.75
**Classification**	allergen
**Cellular Component**	extracellular region
Primary citation	
**PubMed ID**	16984999
**DOI**	10.1073/pnas.0605979103

3.3. Microarray data

In this example, use of *InCHlib* for microarray data analysis is demonstrated. The expression levels of 48,803 genes (rows) were measured for 52 tumour and normal samples (columns) [5]. Original 48,803 genes were compressed to 500 rows. Column metadata distinguish between normal (green) and tumour (magenta) samples. The boxplot on the right, provided by the *Highcharts* library [6], shows expression level distribution in tumour and normal classes.

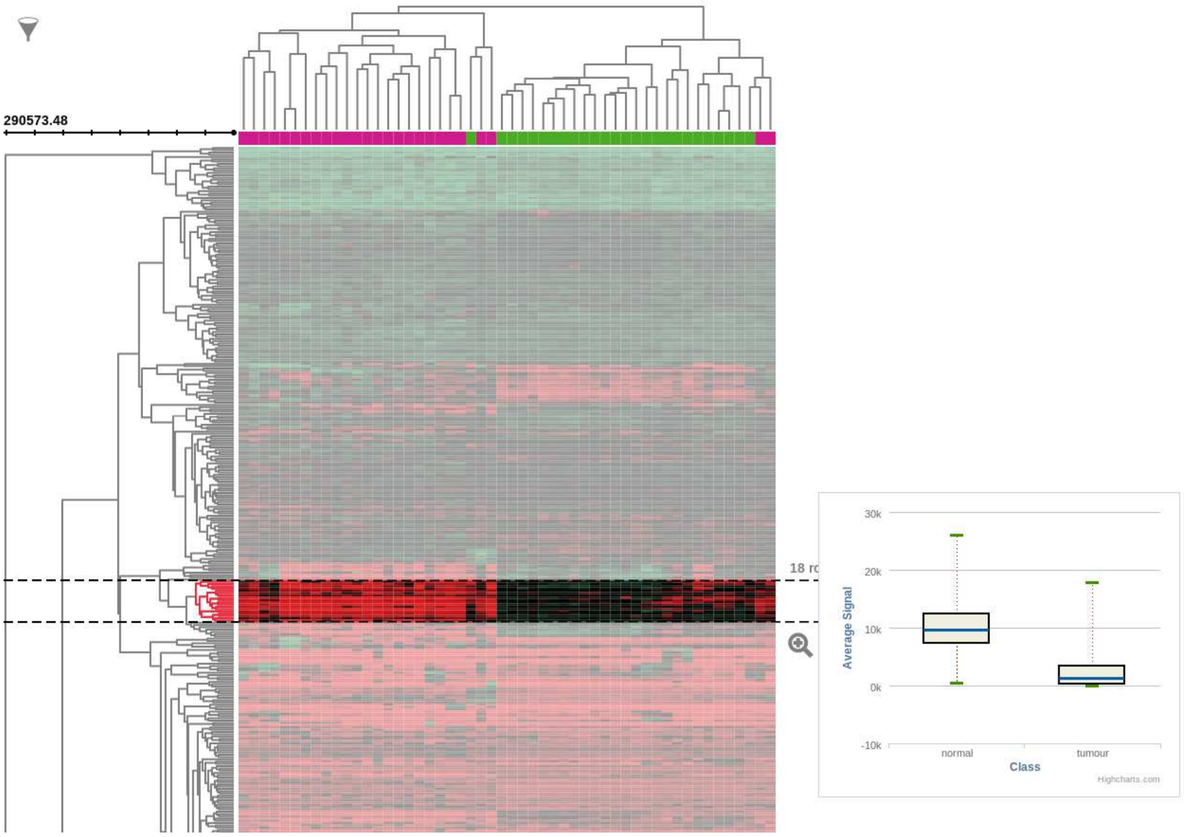

3.4. Scotch whisky distilleries

This example demonstrates the *InChlib* versatility. Data do not originate from a biomedical domain, instead clustering of Scotch whiskies by their twelve taste characteristics was performed. In this example, *InCHlib* is integrated with the *Google map* service.

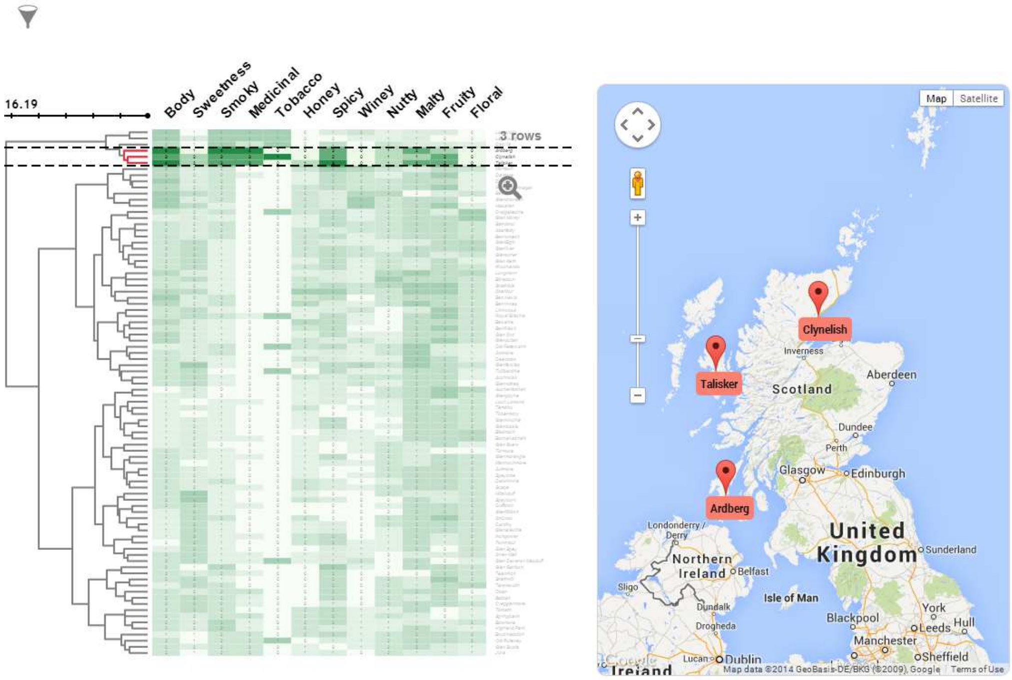

4. References

1. **inchlib_clust Documentation** [http://openscreen.cz/software/inchlib/inchlib_clust]
2. Müllner D: **fastcluster: Fast Hierarchical, Agglomerative Clustering Routines for R and Python**. *Journal of Statistical Software* 2013, **53**(9):1-18.
3. Gaulton A, Bellis LJ, Bento AP, Chambers J, Davies M, Hersey A, Light Y, McGlinchey S, Michalovich D, Al-Lazikani B *et al*: **ChEMBL: a large-scale bioactivity database for drug discovery**. *Nucleic acids research* 2012, **40**(Database issue):D1100-1107.
4. **GLmol Molecular Viewer** [http://webglmol.sourceforge.jp/index-en.html]
5. Kolar M, Szabo P, Dvorankova B, Lacina L, Gabius HJ, Strnad H, Sachova J, Vlcek C, Plzak J, Chovanec M *et al*: **Upregulation of IL-6, IL-8 and CXCL-1 production in dermal fibroblasts by normal/malignant epithelial cells in vitro: Immunohistochemical and transcriptomic analyses**. *Biology of the cell / under the auspices of the European Cell Biology Organization* 2012, **104**(12):738-751.
6. **Highcharts JS** [http://www.highcharts.com/]
