# Supplementary figures and images for: InCHlib – interactive cluster heatmap for web applications
Source: J Cheminform. 2014 Sep 17;6:44. doi: 10.1186/s13321-014-0044-4 (PMC4173117; doi:10.1186/s13321-014-0044-4)

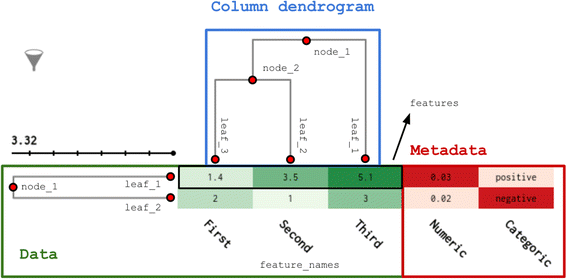

Supplement: Supplementary file 7 — Authors’ original file for figure 1 [file 13321_2014_44_MOESM7_ESM.gif]

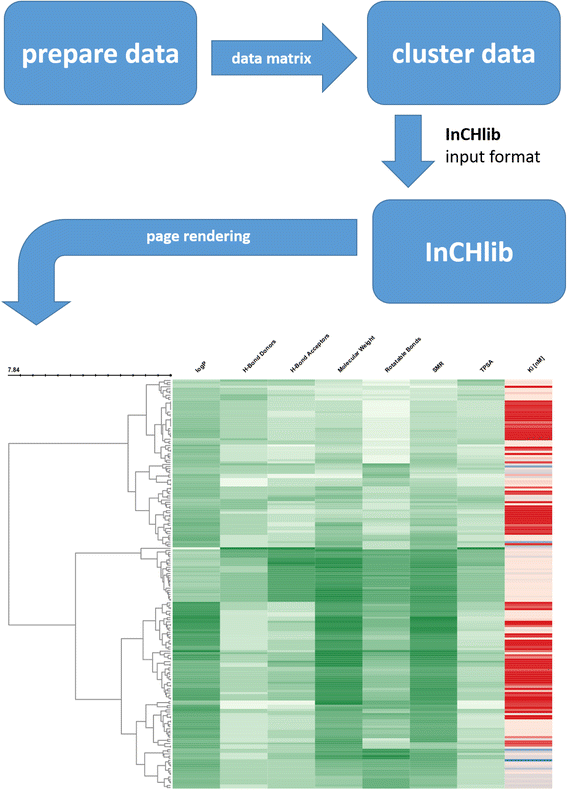

Supplement: Supplementary file 8 — Authors’ original file for figure 2 [file 13321_2014_44_MOESM8_ESM.gif]

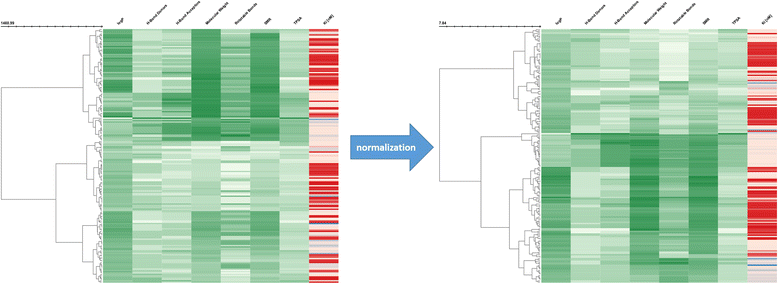

Supplement: Supplementary file 9 — Authors’ original file for figure 3 [file 13321_2014_44_MOESM9_ESM.gif]

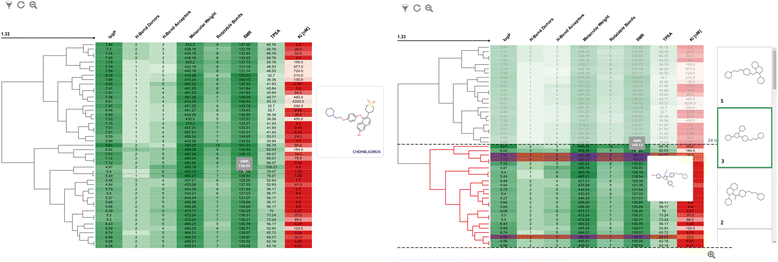

Supplement: Supplementary file 10 — Authors’ original file for figure 4 [file 13321_2014_44_MOESM10_ESM.gif]

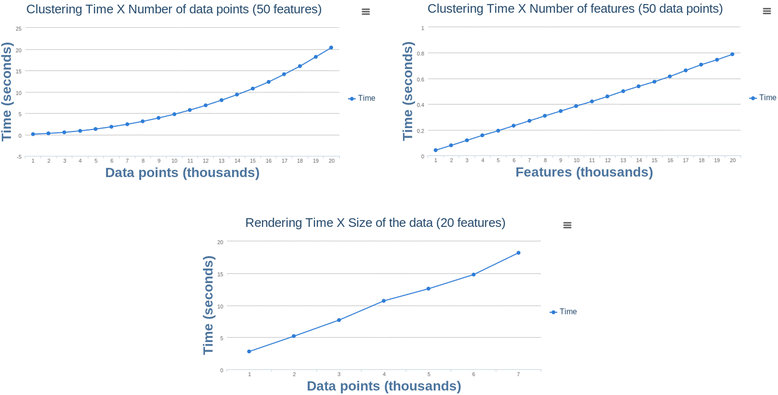

Supplement: Supplementary file 11 — Authors’ original file for figure 5 [file 13321_2014_44_MOESM11_ESM.gif]
